# Paracrine Signaling by Extracellular Vesicles via Osteoblasts

**DOI:** 10.1007/s40610-016-0034-6

**Published:** 2016-02-23

**Authors:** Jess Morhayim, Resti Rudjito, Johannes P. van Leeuwen, Marjolein van Driel

**Affiliations:** Department of Internal Medicine, Erasmus University Medical Center, Wytemaweg 80, 3015 CN Rotterdam, The Netherlands

**Keywords:** Extracellular vesicles, Communication, Transfer, Bone microenvironment, Osteoblasts, Bone metastases

## Abstract

Extracellular vesicles (EVs), spherical bilayered proteolipids, behave as paracrine effectors since they are released from cells to deliver signals to other cells. They control a diverse range of biological processes by transferring proteins, lipids, and nucleic acids between cells and are secreted by a wide spectrum of cell types and are found in various biological fluids. EVs are formed at the plasma membrane or in endosomes and are heterogeneous in size and composition. Increasing understanding of the working mechanisms is promising for therapeutic and diagnostic opportunities. In this review, we will focus on the recent developments in this emerging field with special emphasis on the role of EVs in the bone microenvironment, with a central role for the osteoblasts in the communication with a diversity of cells, including bone metastases.

## Introduction

Communication between cells is required for proper development and functioning of tissues, either via direct interactions or via secreted factors [1]. In the past, these secreted factors included small soluble molecules (neurotransmitters, chemokines, cytokines, hormones) that could behave in a paracrine manner (short distances) or in an endocrine manner (long distances) [2]. A specific route of cell-to-cell communication that has gained more and more attention is communication via extracellular vesicles (EVs). By being studied in diverse biological processes, EVs are discovered now as novel mediators of intercellular communication both in health and disease [3]. EVs are spherical bilayered proteolipids that transfer genetic information incorporated in lipids, proteins, and nucleic acids from one cell to another, thereby influencing the recipient cell function [4, 5•]. They form a heterogeneous group of small particles but are commonly categorized in three main classes: exosomes formed within the endosomal network and released after fusion of multivesicular bodies with the plasma membrane (10–100 nm), microvesicles/microparticles/ectosomes/matrix vesicles produced by outward budding of the plasma membrane (100–1000 nm), as well as apoptotic bodies that are released when dying cells fragment (0.8–5 μm) [2, 5•, 6–8].

The evidence that EVs were not just cellular debris came in 1967 when Wolf and colleagues showed their significance in coagulation [9]. Also in 1967, Anderson and Bonucci discovered the role of EVs as matrix vesicles involved in mineralization of bone extracellular matrix [10, 11]. After the first discovery of the importance of EVs for mineralization of bone, now there is increasing knowledge of the biological role of EVs in many other bone-related processes. In the complex bone microenvironment where many cells reside, there is an important role for EVs to control the intercellular communication.

In this review, we will discuss the current general knowledge on EV isolation and characterization, their molecular composition, biogenesis, and uptake mechanisms. Special focus will be on the role of EVs in the function of osteoblasts, the bone forming cells, in the communication with their microenvironment. This involves not only mineralization but also regulation of stem cell differentiation and the attraction and growth of metastatic cancer cells.

### Isolation and Characterization Methods of EVs

One major challenge in the expanding field of EV research is to improve and standardize methods for EV isolation and characterization. Currently, differential centrifugation is the “gold standard” procedure for EV isolation [12]. In this method, biological fluids or supernatants of cultured cells undergo multiple sequential centrifugations, starting from low speed to remove cellular debris, followed by increasing centrifugal speeds to isolate smaller and less dense particles. Apoptotic bodies and big microvesicles are commonly pelleted at around 10,000 g, whereas small microvesicles and exosomes require high-speed centrifugation ≥100,000*g* [12]. Because of their small size, contamination among EVs has to be tightly controlled in the isolation process. Therefore, serial centrifugation steps are performed to avoid co-isolation of cellular organelles and protein aggregates. EV-depleted serum and/or serum-free medium incubation before EV collection are essential for cell cultures to eliminate EV and protein contaminant from bovine/fetal calf serum [2]. In general, ultracentrifugation approach is extremely sensitive to parameters, such as *g* force, rotor type, duration, and solution viscosity, which cannot be reliably controlled. Other isolation methods for EVs which have been developed to date include ultrafiltration, sucrose density gradient, size-exclusion chromatography, and immunoaffinity capture; however, the efficiency of each method compared to differential centrifugation remains unclear [13].

Most frequently used methods to detect and characterize EVs include biochemical, fluidic, and imaging analyses. While electron microscopy (EM) is commonly used to visualize EV morphology, several groups have shown that the apparent size and shape of EVs observed maybe artifacts from fixation and drying [14, 15]. Cryo-EM provides a better alternative as EV samples are quickly frozen and vitrified, thereby retaining their structure [13]. Furthermore, atomic force microscopy can be used to analyze the morphology of EVs in their native states [2, 16]. Next to imaging methods, Western blot and flow cytometry are also used to study EVs with known vesicle markers [17]. Analyzing smaller sized EVs are difficult with conventional flow cytometry as it cannot distinguish particles <250 nm. This led to the recent development of a high-resolution flow cytometry which could quantify immunolabeled EVs in the range of 100–200 nm [18]. Nanoparticle tracking analysis is another method which allows quantification and determination of size distribution of EVs as small as 50 nm based on their Brownian motion in fluids [16, 19].

### Molecular Composition of EVs

EVs contain a specific composition of nucleic acids, proteins, as well as lipids in a functionally active form. Because of the increasing interest in EV research, public online databases that document the molecular content of EVs are available. These include Vesiclepedia (www.microvesicle.org) [20], EVpedia (www.evpedia.info) [21], and ExoCarta (www.exocarta.org) [22] and are based on proteomic, lipidomic, microarray, and deep sequencing analyses of different EV populations described in the literature. Knowledge on the molecular composition of EVs is pivotal in understanding the relation with cellular origin, biogenesis and interactions with target cells.

The role of EVs in intercellular gene-based communication is supported by the fact that nucleic acids are found to be enriched in EVs. In the late 1990s, EVs co-isolated with viruses were already indicated to contain RNAs [23]; however, it was not until 2006 that the presence of functional RNA in murine stem cell derived-EVs was first described [24]. EVs do not only contain intact mRNA [25] but also miRNA, long noncoding RNA, piwi-interacting RNA, ribosomal RNA, transfer RNA, small nuclear RNA, and small nucleolar RNA [26]. RNA detected in EVs has a predominant size of <70 nucleotides [27]. RNA-inducing silencing complex (RISC), which are involved in miRNA processing, has been detected in EVs, suggesting that EVs may perform cell-independent miRNA biogenesis [28]. Solexa sequencing has identified that genomic DNA fragments are also present in EVs derived from human plasma [29].

In addition to nucleic acids, EVs are also highly abundant in proteins. Most predominantly, cytoskeletal, cytosolic, heat shock, plasma membrane, and vesicular trafficking proteins are found in EVs [2, 30]. Among EV protein, tetraspanins are the most well-described proteins and have been widely used as markers for EVs [31]. These proteins are involved in a broad range of function including EV biogenesis, selection of cargo, as well as binding and uptake by target cells [32, 33]. Tetraspanins may be coupled to chaperones such as heat shock proteins which aid in the sorting machineries of vesicular cargo [34]. Previously described to be specific exosomal markers, tetraspanins CD9, CD63, and CD81 have now also been detected in apoptotic bodies and microvesicles [35]. In addition, CD9, CD63, CD81, CD82, and CD151 are shared among EV groups from various cellular sources, while others are restricted to particular cells, such as Tssc6, CD37, and CD53 in hematopoietic cells [31].

Lipids form the bilayer membrane of EVs providing structure and protecting EV cargo from degradation before they reach their targets. The first studies examining the lipid composition of EVs were conducted on prostate-derived EVs (known as prostasomes) found in seminal fluid [36]. At the moment, the metabolomic analyses on EVs focus on lipids and increasing number of studies are documenting the lipidomics of EVs from various cell lines and biological fluids of multiple species [5•]. EVs are generally enriched in cholesterol, sphingomyelin, phosphatidylserine, and glycosphingolipids, compared to their parent cells [37]. Besides providing structure, EV lipids play pivotal role in vesicular formation, release, and intercellular communication. Cholesterol has been shown to regulate secretion of EVs [38]. Furthermore, bioactive lipids such as prostaglandins and eicosanoids can be transferred by EVs between cells to mediate cell signaling [37, 39]. The specific lipid composition of the EV membrane accounts for their stability to withstand different extracellular environments [5•].

### Biogenesis and Uptake Mechanisms of EVs

At least three distinct mechanisms of EV biogenesis are known, which are exocytosis, direct budding from the plasma membrane, and fragmentation of dying cells, each leading to the release of different EV groups. Reports to date mostly describe exosome biogenesis, and the mechanisms of microvesicle and apoptotic body formation are far less understood [2]. Exosomes, which are the smallest-sized EV class, are released from exocytosis of multivesicular bodies (MVBs) [40]. However, the process by which MVB fuse with plasma membrane and release of exosomes is still unknown. It has been proposed that cytoskeleton and p53 play an important role in these processes, as well as GTPases, such as Rab5, Rab27, and Rab35 [41, 42]. Direct budding from the plasma membrane releases EVs commonly referred to as microvesicles/microparticles/ectosomes. Studies have shown that lipids such as cholesterol and ceramides are important in the release of microvesicles [43]. Apoptotic bodies which are large vesicles are formed from cellular degradation of dying cells. These EVs are commonly phagocytosed immediately to prevent their contents spilling out and cause damage to the surrounding cells. However, they can also escape phagocytosis and target specific cells, but the function of apoptotic body targeting is still under investigation [44].

EV uptake by recipient cells generally depends on the type of target cells. In most cases, EV internalization appears to occur via endocytosis including phagocytosis, micropinocytosis, clathrin-dependent, caveolin-dependent, and lipid raft-mediated endocytosis. However, there appears to be contradictions as to which type of endocytic processes are most important in EV uptake [45]. Besides endocytosis, EV uptake can also occur via membrane fusion. However, direct fusion of EVs with the plasma membrane may be limited to acidic environments, such as those found in tumor microenvironments, as at neutral pH, the rigidity of the membrane prevents fusion [46].

### Role for EVs in Osteoblasts: Mineralization

Since long it is known that in the process of skeletogenesis and bone formation, EVs play an important role. Osteoblasts but also hypertrophic chondrocytes from growth plate cartilage secrete extracellular matrix (ECM) proteins and initiate mineralization via the release of matrix vesicles [47, 48]. Matrix vesicles possess specialized functions that are essential for mineral formation [49, 50]. Mineralizing cells concentrate inorganic phosphate in the cytoplasm and high levels of Ca^2+^ ions in mitochondria prior to mineralization. Released mitochondrial Ca^2+^ and inorganic phosphate form together calcium phosphate at sites of matrix vesicle formation. Matrix vesicles are released from apical microvilli of osteoblasts and/or hypertrophic chondrocytes into the ECM. Once released, the matrix vesicles continue to accumulate Ca^2+^ ions and inorganic phosphate which promotes hydroxyapatite formation. The second phase of mineralization starts with the release of hydroxyapatite crystals from matrix vesicles and the propagation of mineral formation in the ECM [50]. Proteome analysis of matrix vesicles revealed a large number of proteins, like annexins, peptidases, osteoblast-specific factors (alkaline phosphatase, periostin), ion channels, and signal transduction molecules, such as 14-3-3 family members and Rab-related proteins, and proteins that regulate inorganic (pyro)phosphate homeostasis, Ca^2+^-ion homeostasis, intravesicular pH and lipid composition of the EV membrane, all contributing to the understanding of the formation of mineral [50, 51].

Proteomic analysis of EVs derived from different stages of osteoblast differentiation under mineralizing and nonmineralizing conditions revealed that 97 % of the proteins were shared among EVs from mineralizing and nonmineralizing osteoblasts. In the unique group of proteins that were at least fivefold more abundant in EVs from mineralizing osteoblasts were alkaline phosphatase and RNA-binding proteins, in EVs from nonmineralizing osteoblasts was an enrichment of adhesion proteins [52].

Interestingly, matrix vesicles isolated from rat growth plate contained bone morphogenetic proteins, vascular endothelial growth factor, and noncollagenous matrix proteins, confirming also the role for EVs in endochondral bone formation [53].

Studies have focused on the regulatory and mechanistic events supporting mineralization. Transmission electron microscopy showed clustering of matrix vesicles at the plasma membrane extracellular junction prior to their secretion [51]. Thouverey and colleagues confirmed that matrix vesicles originate from apical microvilli of osteoblasts. Cell polarization and apical targeting were required for the incorporation of specific lipids and proteins. Actin-severing proteins such as gelsolin and cofilin and contractile motor proteins such as myosins drive matrix vesicle release from the microvilli to the ECM [50].

The importance of matrix vesicles for mineralization was functionally confirmed by several studies. In bone of hypophosphatasia patients, it was shown that the defects in mineral crystal formation via matrix vesicles led to a decreased level of bone calcification [54]. In human osteoblasts, inhibition of mineralization and altered extracellular matrix composition after in vitro incubation with activin A resulted in a reduced expression of matrix vesicle markers implying deficient or altered matrix vesicles production [55]. Also inhibition of osteoblast mineralization by fibroblast growth factor-2 was suggested to be caused by limiting the capacity of matrix vesicles [56]. In GPM6B-silenced human osteoblasts, which fail to initiate ECM mineralization, EV release of alkaline phosphatase positive EVs was reduced [57]. Stimulation of mineralization by vitamin D treatment increased matrix vesicle secretion from human osteoblasts [58].

### Role for EVs in Osteoblasts: Communication in Bone Microenvironment

Bone and bone marrow form a complex (micro)environment, hosting diverse cell types, among which are hematopoietic and mesenchymal stem cells, endothelial cells, fat cells, cartilage, and nerves. Intercellular communication networks between these cells present are essential for efficient regulation of different processes [2]. It was recently shown that adipogenic RNAs were transferred between adipocytes and osteoblasts via EVs derived from bone marrow adipocytes [59]. It is tempting to speculate that EVs may play a role in the competition between osteoblasts and adipocytes in osteoporosis. Another role for EVs involves directing the differentiation of embryonic stem cells (ESCs). EVs isolated from preosteoblasts were able to deliver genetic material (miRNAs) to undifferentiated ESCs and influenced ESCs differentiation including persistence of pluripotent gene levels and increased neuroectoderm differentiation [60]. This new way to manipulate stem cell differentiation via EVs may improve the potential of using pluripotent stem cell populations for therapeutic applications.

#### Osteocytes and Osteoclasts

Little is known about the role of EVs in the communication of osteoblasts with osteocytes and osteoclasts. Osteocytes form about 90–95 % of the cells in adult bone. These long-lived cells have a stellate morphology with long cytoplasmic extensions (dendrites). They are embedded in bone within the mineralized lacuna, the dendrites extent though canaliculi in the bone matrix [61, 62]. When looking for novel approaches for two- and three-dimensional multiplexed imaging of osteocytes, Kamel-ElSayed and colleagues described as an unexpected finding in the bones of Dmp1-memGFP transgenic mice, adult femurs and mice calvaria, vesicle-like stuctures released from osteocytes with a diameter of 0.5–2 μm [61]. Studies are currently ongoing to determine their composition and function.

Recently, it was shown that osteoblast-derived EVs contain RANKL and could stimulate osteoclast formation [63•], adding a role for EVs in the communication between osteoblasts and osteoclasts as a novel mechanism for bone remodeling. Also, EVs derived from osteosarcoma cells contained pro-osteoclastogenic cargo (MMPs, RANKL, TGF-β, CD9) to increase osteoclastic activity [64].

#### Hematopoietic and Mesenchymal Stem Cells

Hematopoietic stem cells (HSCs) in the bone marrow are multipotent, self-renewing progenitor cells. Differentiated blood cells from the lymphoid and myeloid lineages arise from HSCs. Embryonic stem cell-derived EVs were able to reprogram hematopoietic progenitors: they could expand them as well as increase their pluripotency after horizontal transfer of embryonic stem cell-derived mRNA [24]. By changing the phenotype of HSCs, EVs may contribute to the explanation of the plasticity of stem cells [65].

Mesenchymal stem (stromal) cells (MSCs) in the bone microenvironment are multipotent cells that can differentiate into different cell types, like osteoblasts, chondrocytes, myocytes, and adipocytes [66]. EVs released from MSCs are important in the cell-cell communication involved in tissue regeneration [67]. MSCs release a large amount of EVs containing mRNA with specific properties and selected patterns of miRNAs. When transferred to a recipient cell, the delivery of genetic information alters the gene expression of this cell [68, 69]. Repeated administration of allogenic EVs derived from MSCs does not elicit immune responses as histocompatibility agents are not expressed. MSC-derived EVs used in a model of renal ischemia/reperfusion injury limited acute injury via inhibition of apoptosis/stimulation of proliferation and prevented the development of chronic renal disease [70]. These findings emphasize the importance of EVs in regenerative therapy and/or immunotherapy.

EVs derived from human bone marrow MSCs are also involved in the effects of these MSCs on cancer cell growth and behavior [71]. MSC-EVs inhibited cancer cell growth of HepG2 hepatoma, Kaposi’s sarcoma, and Skov-3 ovarian cancer cell lines. The activation of negative regulators of the cell cycle may explain these effects. MSC-EVs were also capable of inhibiting growth of these cancer cell lines when injected subcutaneously in SCID mice [72]. EVs derived from murine MSCs were shown to significantly downregulate vascular endothelial growth factor (VEGF) in breast cancer cells leading to an inhibition of angiogenesis both in vitro and in vivo [73]. In contrast, Zhu and colleagues showed that bone marrow derived MSCs-EVs promoted tumor growth in vivo but not in vitro. MSCs-EVs enhanced VEGF expression in tumor cells by activating the extracellular signal-regulated kinase 1/2 (ERK 1/2) pathway [74]. This means that EVs from the same source can have opposite effects on different types of cancer, stressing the necessity of new comparative studies. Differentiation stage of MSCs may be of importance in this respect, in particular in the osteogenic direction, since EVs derived from mature osteoblasts enhanced growth of human bone metastatic prostate cells [52].

### Role for EVs in Bone Metastases

**Fig. 1:**
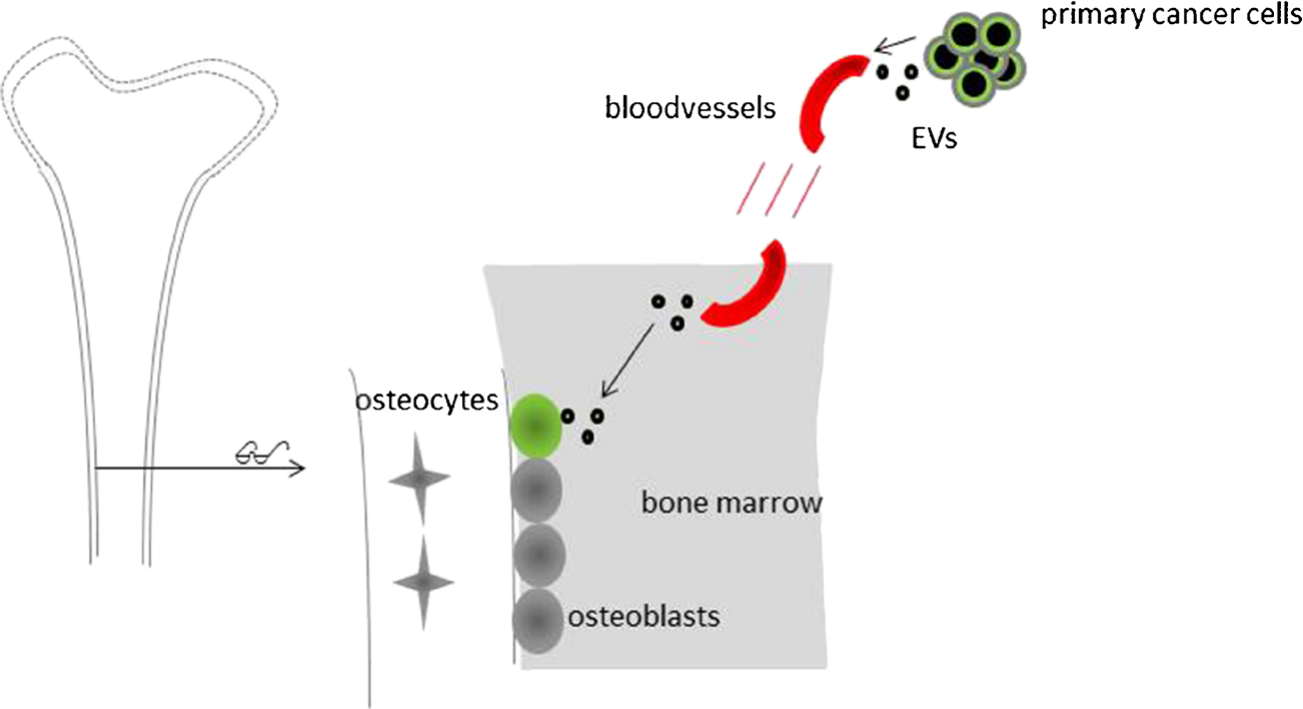
Role for EVs in preparing the metastatic niche in bone. Schematic representation of EVs secreted by primary cancer cells that are transported via the circulation to the endosteal side of the bone marrow. Here, they “educate” the present osteoblasts to prepare a metastatic niche where disseminated cancer cells will attach and grow.

As a nonwanted property, the special milieu of the bone microenvironment provides a fertile soil for many cancers to metastasize to. Especially for patients with breast or prostate tumors, metastatic cells preferentially go to the bone. The consequences of bone metastases are devastating. Severe bone pain, pathologic fractures, hypercalcemia, and spinal cord compression abolish the quality of life. Despite the discovery of many factors involved, no cure has been found yet for bone metastases. The metastatic process is determined by highly specific interactions between disseminating cancer cells and the bone microenvironment [75, 76]. There is strong evidence that EVs secreted by cancer cells may account for angiogenesis and the formation of a premetastatic niche in the bone microenvironment. In a study by Renzulli and colleagues, normal human bone marrow cells were exposed via indirect contact to human prostate tumor cells or isolated EVs from these tumor cells. In the bone marrow cells that were exposed to both prostate cancer cells and their EVs, prostate-specific gene expression was induced [77]. EVs were found to “educate” bone marrow cells toward a pro-metastatic phenotype (Fig. 1). EVs from highly metastatic melanomas increased the metastatic behavior of primary tumors in vivo via permanently “educating” bone marrow progenitors through upregulation of the receptor tyrosine kinase MET in these bone marrow cells [78•]. This property of cancer cell EVs to prepare or educate a pre-metastatic niche in the bone microenvironment demands to search for modes of therapeutic intervention. More knowledge on the processes and mechanisms involved and the possible use of tailored EVs may outsmart niche-preparing cancer EVs [79]. Promising work was performed by Valencia and colleagues. They were able to change cancer EV cargo by re-expression of a single antiangiogenic miRNA (miR-192) and repressed the tumor-induced angiogenesis leading to a reduction in bone metastatic lesions in mice. Changing the miRNA-cargo content in EVs represents a novel mechanism that may strongly influence bone metastases [80••].

## Conclusions

EVs are important players in paracrine signaling in the bone microenvironment. There a many developments ongoing to better understand EV biogenesis and uptake mechanisms. This goes along with improvements in isolation and characterization methods and more knowledge about their molecular composition. In bone, EVs are involved in many processes in the communication of osteoblasts with the bone microenvironment. This encompasses mineralization, but also differentiation of stem cells and interaction with other cells that reside in the bone. In particular, the presence of bone metastatic cancer cells is a pathological condition for which expanding the knowledge on the role of EVs is required to establish also their utilization in prevention and therapy. Overall, the identification and characterization both structural and functional, of EVs opens up novel avenues to regulate bone metabolism as well as the interplay between bone and the cells and tissues within or surrounding bone. In addition, they may evolve to become novel diagnostic indicators for skeletal disorders.
